# Development of Tungsten Oxide Nanoparticle Modified Carbon Fibre Cloth as Flexible pH Sensor

**DOI:** 10.1038/s41598-019-41331-w

**Published:** 2019-03-15

**Authors:** Mamun Jamal, Kafil M. Razeeb, Han Shao, Jahidul Islam, Irani Akhter, Hidemitsu Furukawa, Ajit Khosla

**Affiliations:** 1 Department of Chemistry, Faculty of Civil Engineering, Khulna University of Engineering & Technology, Khulna, 9203 Bangladesh; 2 Micro-Nano Systems Centre, Tyndall National Institute, University College Cork, Dyke Parade, Lee Maltings, Cork, T12 R5CP Ireland; 3 Department of Mechanical System Engineering, Graduate School of Science and Engineering, Yamagata University, Jonan 4-3-16, Yonezawa, Yamagata 992-8510 Japan

## Abstract

A reagent-less pH sensor based on disposable and low cost carbon fibre cloth (CFC) is demonstrated for the first time, where tungsten oxide nanoparticles were grown directly onto the CFC substrate. For comparison purpose, tungsten oxide nanoparticle modified glassy carbon electrode (GCE) was also fabricated as a pH sensor, where hydrothermally synthesized tungsten oxide nanoparticles were drop casted onto the GCE surface. The corresponding equilibrium potential using tungsten oxide/CFC as a pH sensor was measured using open circuit potential (OCP), and was found to be linear over the pH range of 3–10, with a sensitivity of 41.38 mVpH^−1^, and response time of 150 s. In the case of tungsten oxide/GCE as a pH sensor, square wave voltammetry (SWV) was used to measure the shifts in peak potential and was found to be linear with a pH range of 3–11, and a sensitivity of 60 mVpH^−1^ with a potential drift of 2.4–5.0% after 3 hour of continuous use. The advantages of tungsten oxide/CFC and tungsten oxide/GCE as pH sensing electrode have been directly compared with the commercial glass probe based electrode, and validated in real un-buffered samples. Thereby, tungsten oxide nanoparticles with good sensitivity and long term stability could be potentially implemented as a low cost and robust pH sensor in numerous applications for the Internet of Things (IoT).

## Introduction

The pH is a vital parameter influencing numerous number of processes occurring in the aqueous phase or at its interfaces. It affects processes that involve protons or hydroxide ions, their mechanisms, kinetics and their thermodynamics. It also affects the properties of catalysts or reacting substances. Acidity plays an important role in heterogeneous reactions, which is due to the influence of pH on the structure of the interfaces and heterogeneous catalysts. Thereby, in both biological and environmental sectors, pH is a significant parameter that needs to be monitored precisely and continuously. Conventionally, the pH measurement processes take a substantial amount of time with several calibration steps and handling of fragile electrodes. Various metal^1^ and metal oxide based potentiometric pH sensors have drawn much attention in past decades due to their stability against dissolution, independence from cationic interference and low cost fabrication processes^2,3^. Devices for sensing pH were reported using hydrogel membranes, optical fibers, ion sensitive field effect transistors, solid state architectures etc. However, they still face limitations, such as fabrication cost, high energy consumption and design complexity^4–9^. The inspiration of using metal oxides is mostly due to its charge storage capabilities and electro-catalytic, electro-chromic and photo-electrochemical properties. Reducing the size of the materials to the nano level presents many advantages such as a large fraction of surface atoms, high surface energy and high surface to volume ratio, which significantly improves the performances of these materials. In addition, the deposition techniques of these materials need to be cost effective. Electro-deposition of metal oxides is gaining in popularity in industry, which is largely attributed to control over the structure and morphology of deposited materials^10,11^. Additionally, the potentiometric mode is a compatible electrochemical technique for sensing pH in a very usual and portable manner^12^. In recent years, a good number of metal oxides (IrO_2_, WO_3_/IrO_x_ composite, Ta_2_O_5_, RuO_2_, SnO_2_)^9,13–15^ based on their ion-exchanging properties have been studied. In 2009, Fulati *et al*. reported ZnO nanotubes/nanorods as sensing materials^16^ for pH measurements. Vieira *et al*. in 2012, reported the detection of pH as extended gate FET with Layer-by-Layer Films of Dendrimers/TiO_2_ Nanoparticles^17^. Among these oxide materials, WO_3_ is found to be a promising material for sensing pH because of its good catalytic activity, low cost, appropriate physical structure and morphologic control of the nano-structures, stability, reversible conductivity change with highly sensitive, selectivity and biocompatible characteristics^4,5,18^. For developing tungsten oxide modified surfaces, a number of methods can be implemented, such as electro deposition from a precursor solution, anodic oxidation and drop-casting of already synthesized nanoparticles. In 1987, Natan *et al*.^19^ reported a WO_3_ based pH sensor, which was the first micro-electrochemical transistor device that can be turned on at negative values of gate voltage relative to saturated calomel reference electrode, and also showed that tungsten oxide can sense the pH changes through a large, reproducible response of the electric current in real time. However, the high surface area WO_3_ was applied principally as a gas sensor rather than fully demonstrated as pH sensor^20,21^. Lidia *et al*.^22^ has recently reported a WO_3_ based pH sensor, where gold supported tungsten oxide electrode was used as the working electrode with a linearity of pH 5 − 9, and a sensitivity of 56 mV/pH. A tungsten trioxide based pH sensor was reported by Jung *et al*.^23^, which exhibits a linear behaviour in the pH range 1 to 7. In 2012, Guidelli *et al*.^24^ reported a pH-EGFET sensors that was based on V_2_O_5_/WO_3_ mixed oxide films which is continually re-usable. In 2014, Drensler *et al*.^25^ investigated the relation between pH sensing properties with the various preparation routes of tungsten oxide films and made a link with the possible (nano) architecture of the sensing system. In 2015, Campos *et al*.^26^ studied the sensitivity of WO_3_ based pH sensors to compare the extended–gate field effect transistor (EGFET) and cyclic voltammetric (CV) method. In 2018 Pankaj *et al*.^27^ developed hybridized WO_3_ nanostructure based pH sensors that can be applied in food safety through electrochemical detection of harmful dyes. As the requirement for pH sensors is essential in multi-purpose applications, a different design with enhanced flexibility is required, which cannot be provided by conventional solid state-type electrodes^28^. pH sensors based on silicon nitride films^29^, paper^30^ and polyimide surfaces^13^ are reported to be flexible. However, it is quite challenging to address factors like resistance to mechanical stresses, surfaces with diverse planes, and reduction to miniaturized sizes^14,31^. Moreover, in the majority of cases, the sensitivity property of WO_3_ was mainly reported for the applications in solid state pH sensor, and a very limited number of cases have been fully exploited as flexible or conformable pH sensing layers.

In this work, we grown tungsten oxide using a hydrothermal method directly onto CFC and have separately synthesized tungsten oxide hydrothermally and drop casted onto commercially available GCE. The sensing capabilities of both systems were investigated and validated in respect of static and dynamic properties such as calibration, stability, response time, as well as sensitivity in oxygenated and deoxygenated environment. We have found both WO_3_/CFC (direct growth) and WO/GCE exhibit an excellent linearity with better sensitivity and stability in both acidic and alkaline environment.

## Results and Discussion

### Structure and morphology characterization

**Figure 1:**
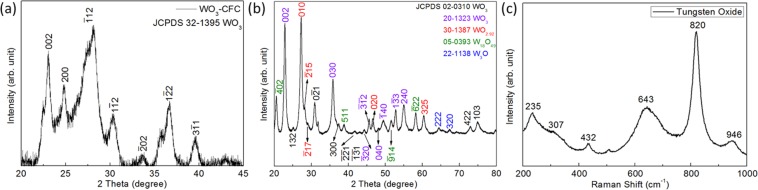
XRD of (**a**) WO_3_/CFC and (**b**) WO nanoparticles, (**c**) Raman spectra of WO nanoparticles.

Figure 1a shows the XRD pattern of the crystal structures and phases of WO_3_/CFC and tungsten oxide nanoparticles. The high crystallinity of the materials was revealed by the intense and narrow diffraction peaks. The intense diffraction peaks located at 23.06°, 24.87°, 28.12°, 30.42°, 33.82°, 36.7°and 39.5° correspond to the tungsten oxide anorthic phase (JCPDS No. 32–1395). The purity of the material is confirmed by the lack of other peaks in the XRD patterns. The average crystal size of tungsten oxide was determined from the Scherer equation, $D=\frac{K\lambda }{\beta \,\cos \,\theta }$, where *K* is a dimensionless shape factor of the crystallite (a typical approximation is ~0.9), *λ* is the wavelength of the X-ray used (*λ* = 1.5406 Å), *β* is the full width at half maximum (FWHM) of the diffracted peak (in radians) and *θ* is the Bragg angle (in degree). The average crystallite size of tungsten oxide was found to be ~5.74 nm.

Figure 1b shows the XRD pattern of the tungsten oxide (WO) nanoparticles sample. All the peaks can be indexed to the planes corresponding to different crystal structures of tungsten oxides. WO_3_ and WO_2.92_ are the major components of WO nanoparticles. However, peaks from W_18_O_49_ and W_3_O are also apparent. Therefore, it can be concluded that the as prepared sample has different tungsten oxidation states. WO nanoparticles were further analysed using Raman and the Raman spectrum is shown in Fig. 1c. The strong peaks at 643 and 820 cm^−1^ correspond to the O-W-O stretching band.^11^ The peak at 235 and 307 cm^−1^ represents the bending vibration δ (O–W–O). The peak at 432 cm^−1^ is assigned to W^V^ = O bonds. The peak at 946 cm^−1^ can be attributed to the terminal -W^VI^ = O bond. The Raman spectrum further confirms the W^VI^ is the main component of WO nanoparticles^11^.

**Figure 2:**
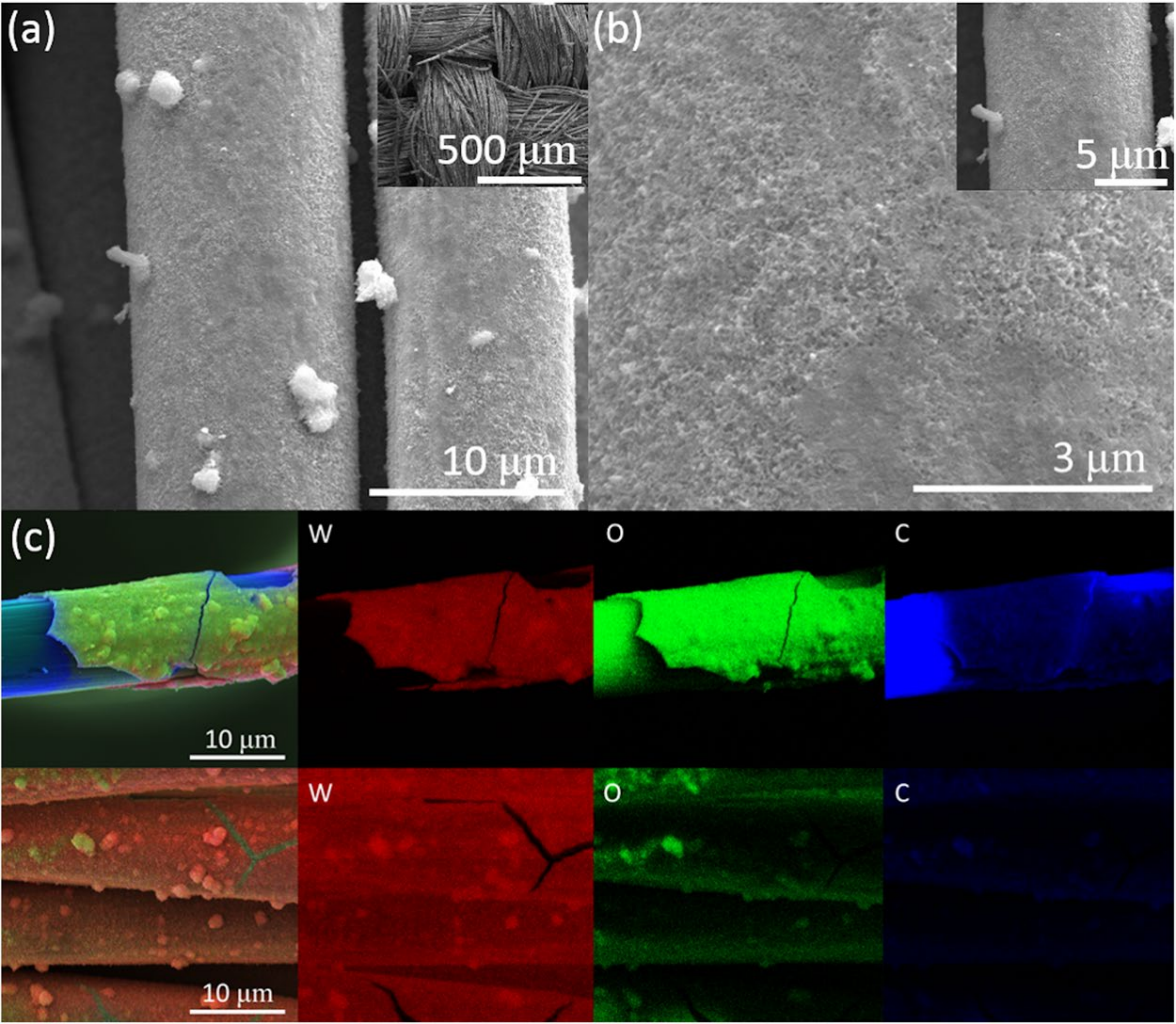
(**a**,**b**) SEM images at different magnificationsof WO_3_/CFC, (**c**) EDX elemental mapping for W, O and C elements.

**Figure 3:**
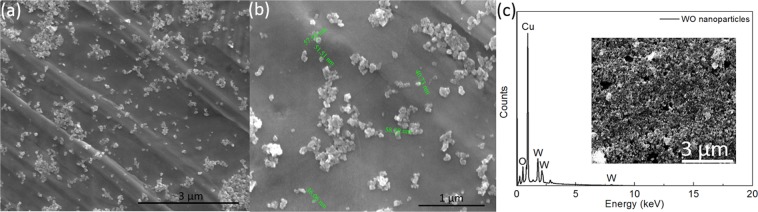
(**a**,**b**) WO nanoparticles at different magnifications, (**c**) EDX of WO nanoparticles. (Cu was detected from the copper tape).

The surface morphology and microstructure of the WO_3_/CFC and WO nanoparticles were observed by scanning electron microscopy (SEM) (Fig. 2a,b). Low magnification SEM images (Fig. 2a) of WO_3_/CFC confirm the homogeneous growth of a tungsten oxide nano layer over the CFC. High magnification SEM images (Fig. 2b) show the porous structure of the WO_3_, which offers a high surface area. EDX elemental mapping (Fig. 2c) clearly indicates the existence of the elemental W and O containing in the WO_3_/CFC sample. SEM images of WO nanoparticles at different magnifications are presented in Fig. 3a,b. An average value of the particle size is 50 nm. According to the EDX analysis, Fig. 3c, the average content of Tungsten (W) and Oxygen (O) is 24.5% (atomic percentage) and 75.5%, which confirms that WO_3_ and WO_2.92_ are the major constituents of the tungsten oxide nanoparticles and can be corroborated with our XRD data, which showed high intensity diffraction peaks for these compositions.

**Figure 4:**
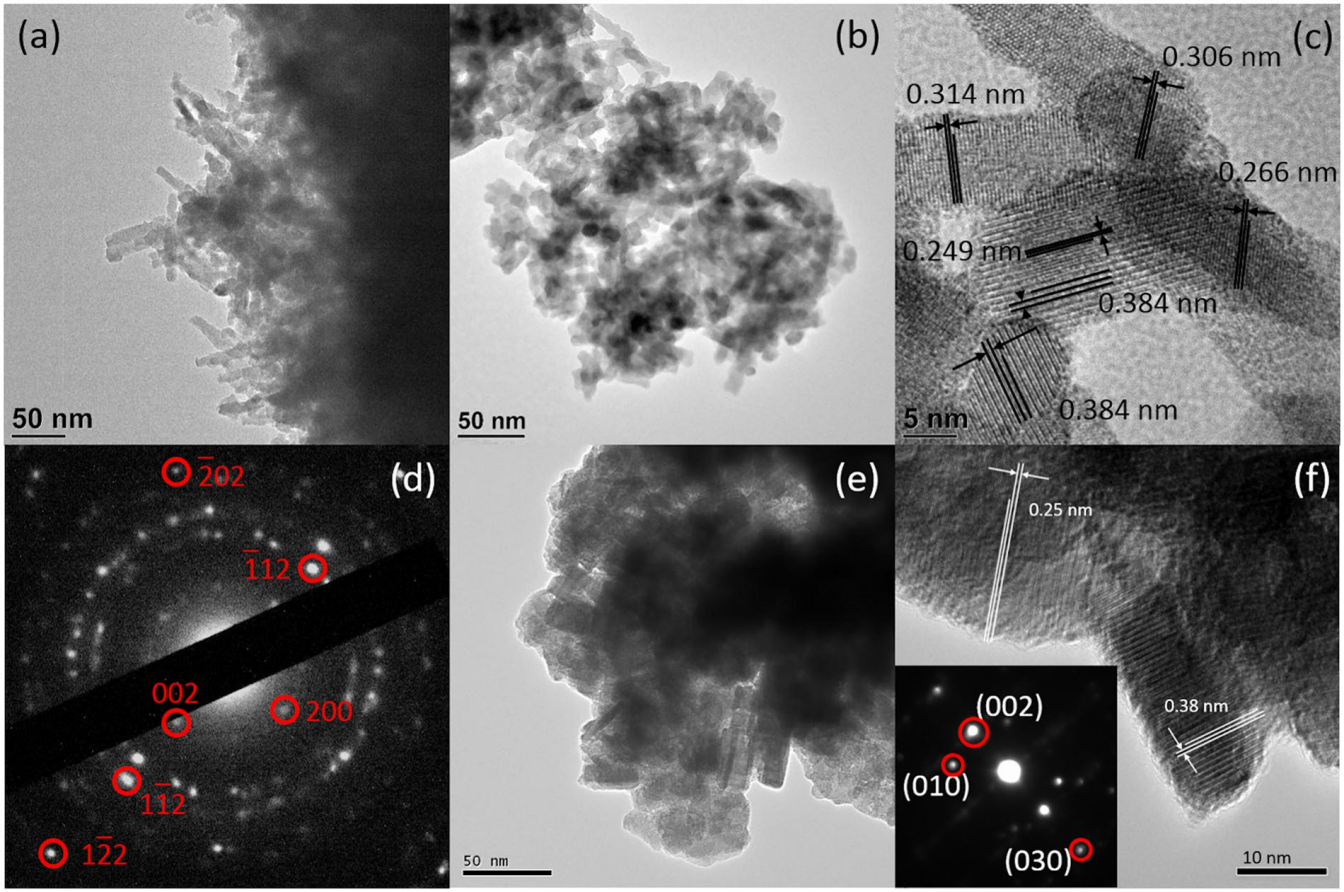
(**a**,**b**) HRTEM image of WO_3_/CFC at different magnifications, (**c**) measured lattice space and (**d**) the corresponding SAED pattern of WO_3_/CFC sample, (**e**) HRTEM image of WO nanoparticles, (**f**) measured lattice space and the corresponding SAED pattern of WO nanoparticles.

Further inspection of both tungsten oxide samples was carried out using transmission electron microscopy (TEM) analysis. The thickness of the tungsten oxide nano layer is 100–200 nm (Fig. 4a), measured from the scratched off samples of WO_3_/CFC, as well as from SEM. The observed average size of tungsten oxide particles is ~5 to 10 nm (Fig. 4b). Figure 4c shows the d-spacing of 0.249, 0.266, 0.306, 0.314 and 0.384 nm, which assigned to the planes of tungsten oxide (32–1395). The selected area electron diffraction (SAED) pattern in Fig. 4d confirms the growth of pure WO_3_ over CFC.

The TEM images, measured lattice spacing and the corresponding SAED pattern of WO nanoparticles are shown in Fig. 4e,f. As observed, the average size of the particle is around 50 nm, which is the same as the particle size determined from SEM. The measured lattice space and the corresponding SAED pattern are all assigned to WO_3_ (20–1323) and WO_2.92_ (30–1387) phases. All the TEM results are consistent with the XRD analysis, which confirm the growth of two different tungsten oxide samples.

### pH sensitivity and reproducibility

**Figure 5:**
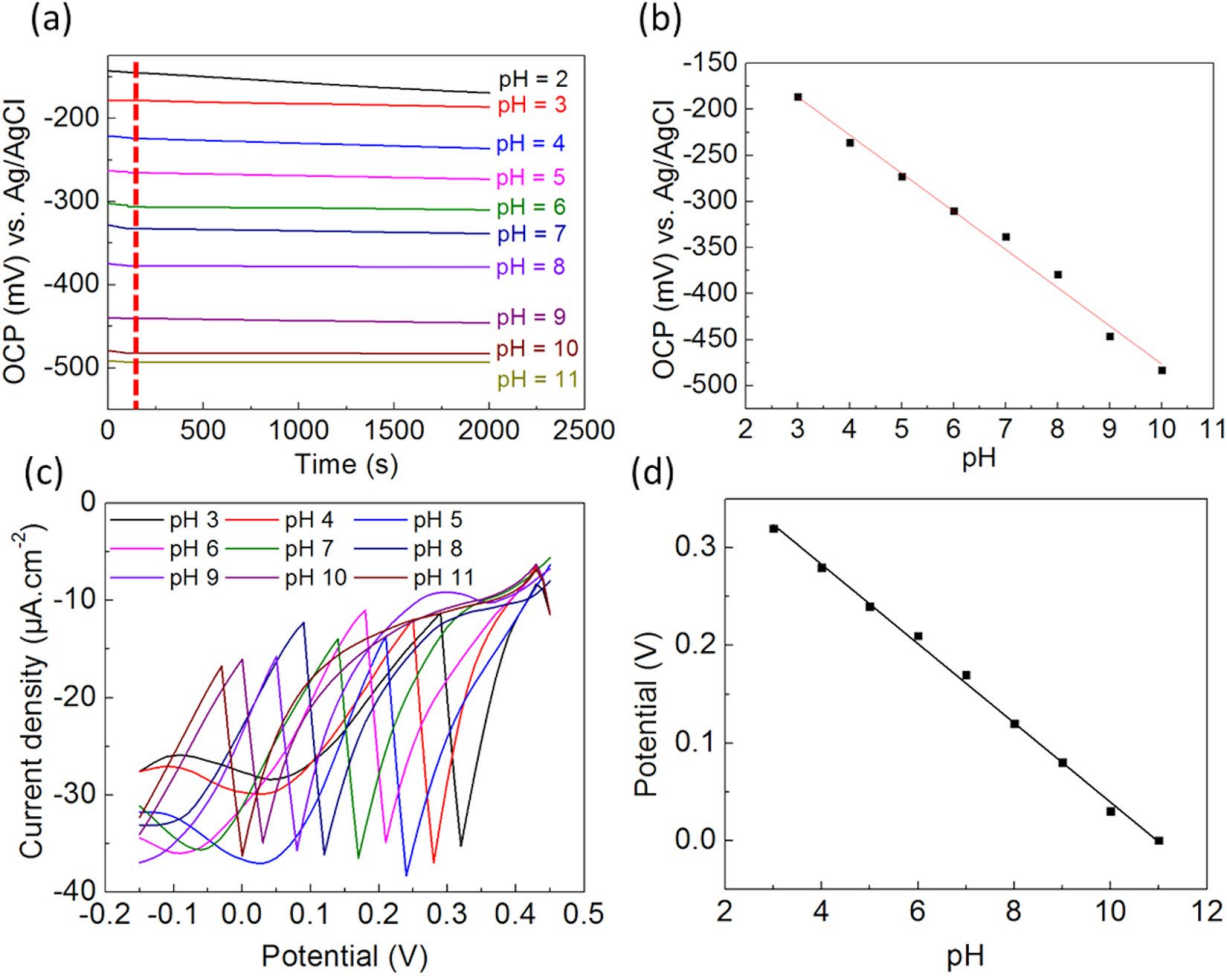
(**a**) Potential response of WO_3_/CFC at different pH (2–11) in 0.1 M buffer-solution and (**b**) the corresponding linear relation plot of pH (3–10); (**c**) SWV of WO/GCE obtained in 0.1 M buffer at different pH (3–11) and (**d**) the corresponding linear relation plot.

The WO_3_/CFC and WO/GCE were tested using OCP and SWV^6^ respectively in buffer solutions with a pH range from 2 to 11 (Fig. 5). Both SWV and OCP can be used to obtain these changes in potential with the change in H^+^ concentrations. Although, finding peaks or position of peak potential in SWV is more dependent on the nature of electrolytes, this is not the case for OCP. Therefore, in both SWV and OCP, tungsten oxide based sensing platform would provide accurate results on sensing pH.

Generally, pH is the measure of H^+^ ions concentration in the solution, which can be related to the redox potential according to the following Nernst equation (equation 1)^13^. Here, E^0^ is the standard electrode potential, R is the molar gas constant, T is the absolute temperature and F is the Faraday’s constant.

1 $$E={E}^{0}-(\frac{2.303\times RT}{F})={E}^{0}-0.05916\,pH$$

The pH response of WO_3_/CFC electrode was investigated in 0.1 M phosphate buffer solution (Fig. 5a,b). The corresponding equilibrium potential was measured by changing the value of pH from 2 to 11, and the response time was found to be only ~150 s. However, it should be noted that the material is not sensitive in highly acidic or alkali environment. WO_3_/CFC showed a pH sensing capability in the range of pH = 3–10 (Fig. 5b) with a sensitivity of 41.38 mVpH^−1^, which is ~70% of the Nernst response (59.2 mVpH^−1^).

On the other hand, WO/GCE electrode was tested with SWV in 0.1 M phosphate buffer in the pH range of pH = 3–11 (Fig. 5c,d). SWV was performed, as it is found to be more sensitive and much faster in speeds compared to other voltammetric techniques.^6^ In the SWV (Fig. 5c), a clear single reduction peak was obtained, which was due to the presence of electro-active species in the electrolyte. The increase of the solution pH results in peak potential shift towards the more negative potential value. The sensing mechanism of WO/GCE is plausibly attributed to the redox reaction that involves the incorporation of cations and electrons simultaneously into the nano-structure of tungsten oxides. Hence, a tungsten oxide bronze (H_x_WO_3_) is formed which shows higher conductivity when compared to tungsten oxide (equation 2).

2 $$W{O}_{3}+x{e}^{-}+x{H}^{+}\to {H}_{x}W{O}_{3}$$

The above reaction involves consumption of electrons (e^−^) and protons (H^+^), where the number of electrons and protons is denoted by *x* in equation (2) The mechanism of this reaction can be described by means of a small polaron transition (W^5+^ site formation) for an amorphous film and free electron scattering (Drudelike) in the case of a crystalline films. For both mechanisms in the crystalline or amorphous films, a shift in the peak potentials is attributed to electron localization- delocalization into the d-orbitals of W^8-10^. Therefore, the measured value of the potential relies on the pH and a linear correlation has been obtained between these two parameters, which signifies Nernstian behaviour. In these circumstances, all the formed surface charges occur due to the redox process, demonstrating with good performance of the fabricated sensors. Both SWV and OCP can be used to obtain these changes in potential with the change in H^+^ concentrations. Although finding peaks or position of peak potential in SWV is more dependent on the nature of electrolytes, this is not the case for OCP. Therefore, in both SWV and OCP, a tungsten oxide based sensing platform would provide accurate results in pH sensing.

The pH sensor fabricated in this work (Fig. 5d) is revealed to show a mean sensitivity value of 60 ± 0.01 mV/pH and this value close to the theoretically determined value. The correlation coefficient (R^2^) is ~0.99, which confirms a high sensitivity of the WO nanoparticles to changes in the concentration of H^+^ ions in the solution, which is involved in redox process occurring in the system. Interestingly, the sensitivity of our pH sensor compares favourably with recent reports tabulated in Table 1. According to the best of our knowledge, no other work has demonstrated the implementation of WO_3_/CFC and WO/GCE as sensing platform for detecting pH, which is environmental friendly and fabricated with earth abundant materials as compared to other available systems.

**Table 1:** Comparison of different electrochemical pH sensors.

Electrode	Sensitivity mV/pH	Drift %	pH range	Reference
WO_3_/CFC	41.38	<11.9	3–10	This work
WO/GCE NPs	60 ± 0.01	2.4–5.0	3–11	This work
Gold/WO_3_ NPs	−56.7 ± 1.3	—	5–9	^7^
AQ–Fc/AuNAE	70	1–3	2–11	^1^
AQ–Fc/GCE	52	<5	3–8	^17^
Gold/CuO NFs	28	—	2–11	^18^
AQ–CNT/GCE	51	1.4	3–10	^19^
AQ– Sulfonate/GCE	38	2–3	2–10	^20^
Thick Film/RuO_2_	30	—	4–10	^21^

### Electrochemical characterization of WO_3_/CFC and WO/GCE in the presence and absence of oxygen

**Figure 6:**
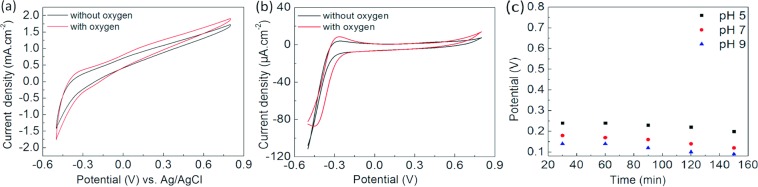
CVs at presence/absence of oxygen of (**a**) WO_3_/CFC and (**b**) WO/GCE, (**c**) Drift of WO/GCE electrode.

The dissolved oxygen concentrations are affected by many factors, such as temperature, pressure, total dissolved substances, amount of pollutions, salinity, etc. In another word, it is hard to adjust the oxygen concentration every time before the pH measurement. Thereby, it is necessary to evaluate the fabricated pH sensors in the presence and absence of oxygen environment to ensure the reliability. To obtain the de-oxygenated environment, the buffer solution was flushed by nitrogen for 20 minutes with a cap on the top so that O_2_ cannot enter the solution during the experiment. CV responses in Fig. 6a,b show the behaviour of both sensors in oxygen containing and oxygen free phosphate buffer solutions. There are no significant changes in the potential both in the oxygenated and deoxygenated environments. However, for both sensors there is a small increase in reductive current in the presence of dissolved oxygen in the solution. Thereby, it is evident that these sensors can be applied to measure the pH, irrespective of O_2_ concentration of the solutions.

### Drift and stability measurement

Due to high sensitivity, WO/GCE has been tested for the drift and stability measurement. SWV was recorded for buffer solutions with three different pH of 5, 7 and 9 as a representative of the three conditions: mildly acidic, neutral and mildly basic. Data have been recorded for three hours with an interval of 30 minutes in each buffer solution (Fig. 6c). The data show that up to 90 minutes may be required for stabilization of the sensor, which can depend on the pH of the system. Within three hours, the potential drift in a buffer solution with neutral pH was found to be 33 mV. Similarly, potential drifts of 24 and 50 mV were observed in acidic and basic media, respectively. The largest drift can be observed at pH 9 among the three buffers (Fig. 6c), which represents a deviation of 5% from the peak potential. In order to determine the stability of the sensors, the signal was recorded continuously for 7 days thereafter, where sensitivity remained up to 95%.

Response time of WO_3_/CFC sensor was measured in buffers with different pH. The OCP is the voltage measured when no current flows through the cell. Generally, we propose a tiny potential variation less than 5 mV within 100 seconds as a stable signal. It can be clearly seen in Fig. 5a that the potential became steady after 150 s in different pH. These response time are found to be higher in compare to other reported metal oxide based pH sensors^32,33^. This may be attributed to the structure of the sensor platform, such as porosity and film thickness of the sensing layer^34^, as it requires higher time to reach equilibrium. It is important to mention that this sensor can respond to commercial buffers and to real sample solutions with a complex mixture of different ions. Therefore, it allows us to confirm that WO_3_/CFC has a good selectivity to protons. However, further work is needed to improve the surface interface, which should reduce the response time of this CFC based pH sensor.

### Real sample test

**Figure 7:**
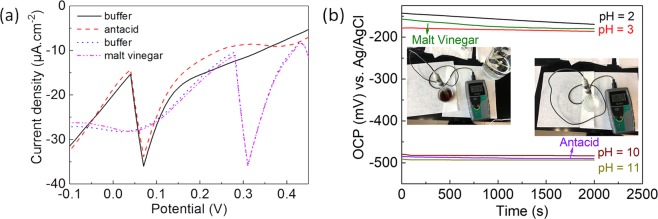
(**a**) SWV obtained in buffer (pH = 4 and 9) and “real” unbuffered samples for malt vinegar and antacid using WO/GCE; (**b**) OCP obtained in buffer (pH = 2, 3, 10, 11) and “real” unbuffered samples for malt vinegar and antacid using WO_3_/CFC.

There is little or no evidence of applying metal oxide based pH sensor in real and unbuffered samples to measure pH^4,18^. In this work, we have validated WO/GCE and WO_3_/CFC as pH sensor against the laboratory standard glass membrane pH electrode in the real samples: malt vinegar and antacid. The electro-analytical signal obtained by WO/GCE using SWV (Fig. 7a), showed that the pH value obtained for malt vinegar (AFP) and antacid (Beximco) are 4 and 9 respectively. pH value for both samples was verified using commercial glass pH sensor, which gives a value of 4 and 9 for the vinegar and antacid. Coincidently, WO_3_/CFC showed 2.6 and 10.5 for malt vinegar (Sarsons) and antacid (Rennie) (Fig. 7b), which were close to the values (2.59 and 10.62) received from the laboratory standard glass pH electrode (Fig. 7b insets, respectively). These results demonstrate that there is a huge potential for this approach for developing a hand-held, portable, voltammetric pH sensor using tungsten oxide based electrodes.

## Conclusions

In this study, we have developed two cost effective pH sensors using WO_3_/carbon fibre cloth and tungsten oxide nanoparticle modified glassy carbon electrode. A flexible sensor based on WO_3_/CFC (direct growth) exhibit an excellent linearity from pH 3 to 10 with an acceptable sensitivity of 41.38 mV/pH, while a wider range from pH 3 to 11 was found using WO/GCE sensor with better sensitivity of 60 ± 0.01 mV/pH. Both sensors showed excellent behaviour in either acidic or alkali environment, which will expand the applications of these pH sensors. The electrodes were effective under both ambient and de-oxygenated conditions, which can further expand their potential applications as pH sensing devices in oxygen-free environments.

In conclusion, the results of this work show that, tungsten oxide is a highly promising material for a stable, sensitive pH sensor. Both WO_3_/CFC and WO/GCE have the potential to replace expensive noble metal based sensors in future widespread applications of wireless sensor nodes (WSN) for the deployment of IoT. Currently we are working on improving the sensitivity of WO_3_/CFC by improving the properties of CFC. In addition, we are also examining if there is any impact of the phase of tungsten oxide on the sensing capabilities.

## Methods

### Materials

Sodium tungsten (Na_2_WO_4_·2H_2_O), sodium chloride (NaCl), hydrochloric acid (HCl) were purchased from E-Merck, Germany. The purchased phosphate buffer salts, sodium hydroxide (NaOH), Nafion and chitosan were obtained from Sigma-Aldrich, Ireland. Malt vinegar (Sarsons) and antacid chewable tablet (Rennie) were purchased from Ireland, while malt vinegar (AFP) and antacid chewable tablet (Beximco) were purchased from Bangladesh. All the chemicals were of high purity (analytical grade) and were used as soon as received. Buffer solutions of different pH were prepared using sodium phosphate salt, sodium hydroxide and hydrochloric acid.

### Synthesis of WO_3_ on CFC

Carbon fibre cloth (3 × 3 cm^2^) was treated in 3 M HCl with ultrasonication for 10 minutes, followed by washing with ethanol and deionized water (DI water) for 15 min, then left overnight for drying to be used as substrate. To synthesize WO_3_ nanoparticles hydrothermally, 1 g Na_2_WO_4_·2H_2_O was dissolved first in 80 ml DI water with 1 g NaCl (as structure directing agent). Then the solution was acidified by drop wise addition of 6 M HCl with stirring until pH value reached to 2.0. Finally, the solution was transferred to 100 mL Teflon-lined stainless steel autoclave (content the pre-treated CFC substrate) and installed in an oven. The synthesis conditions of WO_3_ were fixed at 180 °C for 24 h and thereafter a natural cooling to room temperature. DI water, ethanol and acetone were used three times to wash the synthesized material. Finally, the sample was dried for 12 h in the oven at a temperature of 60 °C to obtain a white layer on CFC The sample was weighted before and after synthesizing and the typical mass of tungsten oxide layer was found to be ~0.3 mg/cm^2^. The growth can be explained by the following chemical reactions (equations 3 and 4):

3 $$2N{a}^{+}+W{O}_{4}^{2-}+HCl\to {H}_{2}W{O}_{4}+2NaCl$$

4 $${H}_{2}W{O}_{4}\to W{O}_{3}+{H}_{2}O$$

### Fabrication of WO/GCE

**Figure 8:**
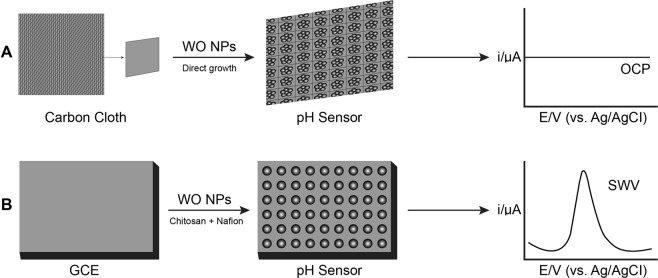
Schematic showing fabrication of tungsten oxide modified (**A**) CFC and (**B**) GC electrode based pH sensor.

To synthesize the tungsten oxide powder, hydrothermal method was used as following: 0.4 g of Na_2_WO_4_·2H_2_O was dissolved in 8 mL of DI water, which was mixed with 0.15 g of NaCl (a structure directing agent) followed by acidification with 0.8 mL solution of 6 M HCl. Finally, the solution was shifted to 40 mL Teflon-lined stainless steel autoclave and installed in an oven. The synthesis conditions of WO nanoparticles were fixed at 180 °C for 1 h and thereafter a natural cooling down at ambient temperature. The synthesized material was separated from the precursor solution by centrifuging at a rpm of 3000 for around *2 *minutes and then washed with DI water (three times). Finally, the product was undergone drying in an electric oven for at least 8 h at a temperature of 60 °C to obtain a powder like nanomaterials.^14^ GCE is used in this study as a substrate and modified using WO nanoparticles. The modified electrode was prepared by a facile casting technique^10^. Prior to the surface coating, the active part of the GCE was cleaned and polished well using 1.0 and 0.3 µm thick alumina powder and finally it was rinsed with DI water, followed by successive ultra-sonication in ethanol solution and DI water. A mixture of tungsten oxide nanoparticles, chitosan and Nafion was drop casted on to the polished active surface of the GCE, and was left overnight for drying at room temperature. The schematic of the fabrication process is shown in Fig. 8.

### Material characterizations

The crystalline structures of the WO_3_/CFC and tungsten oxide nanoparticles were characterized by X-ray diffraction (XRD) (Oxford Instruments INCA energy system, XRD Philips PW3710-MPD diffractometer with Cu Kα radiation, λ = 1.54 Å) and Raman spectra (model: Renishaw (RA100) via confocal Raman Microscope at 514.5 nm excitation laser wavelength). FEI QUANTA 650 field-emission scanning electron microscope (FE-SEM) with an energy dispersive X-ray spectroscopy (EDX Oxford Instruments INCA energy system) and high resolution transmission electron microscope (JEOL HRTEM-2100 at 200 kV) were applied to examine the surface morphology and elemental composition of the fabricated materials.

### Electrochemical Measurements

A classic three electrode setup was used to evaluate these two potentiometric pH sensors through their catalytic action in PBS buffer solutions of various pH (2–11). Platinum (Pt) wire as an auxiliary electrode and Ag/AgCl was used as reference electrodes. CHI 660 C electrochemical workstation and a Dropsens potentiostat (µStat 8000, Spain) were used for all the electrochemical measurements, including OCP, SWV and CV. For conducting SWV measurements, the parameters were set to a step height of 10 mV with a pulse width of 20 mV and a frequency of 50 Hz. Orion commercial glass membrane based pH sensor was used for accurate determination of the pH of prepared buffer solutions. For real sample test, malt vinegar was used directly and antacid chewable tablet was dissolved in 200 ml DI water, and stirred for 1 hour before use.
